# Ball-Mill-Inspired Durable Triboelectric Nanogenerator for Wind Energy Collecting and Speed Monitoring

**DOI:** 10.3390/nano13050939

**Published:** 2023-03-05

**Authors:** Qinghao Qin, Xia Cao, Ning Wang

**Affiliations:** 1 Center for Green Innovation, School of Mathematics and Physics, University of Science and Technology Beijing, Beijing 100083, China; 2 Beijing Institute of Nanoenergy and Nanosystems, Chinese Academy of Sciences, Beijing 100083, China

**Keywords:** triboelectric nanogenerator, wind energy, low wear, rotation speed, self-powered sensor

## Abstract

Triboelectric nanogenerators have attracted extensive attention in energy harvesting due to its light weight, low cost, high flexibility, and diversity of function. However, deterioration in terms of mechanical durability and electrical stability of the triboelectric interface during operation, which are the results of material abrasion, severely limits their practical applications. In this paper, a durable triboelectric nanogenerator inspired by a ball mill was designed by using metal balls in hollow drums as carriers for charge generation and transfer. Composite nanofibers were deposited onto the balls, increasing the triboelectrification with the interdigital electrodes in the inner surface of the drum for higher output and electrostatic repulsion to each other for lower wear. Such a rolling design cannot only increase mechanical durability and maintenance convenience, where the filler can be easily replaced and recycled but also collect wind power with the decreased wearing of materials and sound efficiency in comparison with the typical rotation TENG. In addition, the short circuit current shows a strong linear relationship with the rotation speed in a wide range, which can be used to detect wind speed, thus showing potential applications in distributed energy conversion and self-powered environmental monitoring systems.

## 1. Introduction

With the depletion of fossil energy and concerns about environmental pollution, innovative energy harvesting devices, such as triboelectric nanogenerators (TENGs), are drawing increasing attention because they can scavenge renewable energies from our ambient environment on the basis of coupled friction electrification with electrostatic induction and conform to the trend of the times [1,2,3,4]. Nowadays, due to its high instantaneous output power, a broad selection of available materials, an eco-friendly and inexpensive fabrication process, and various working modes customized for various application scenarios, TENG is advancing for use in self-powered sensors, energy harvesters and other sustainable and intelligent systems with more comprehensive and rational designs of TENGs for various applications [5,6,7,8,9].

Extracting energy from wind sources, which contains a reasonable proportion of the current renewable energy structure, has attracted extensive attention in the TENG’s community, which may complement conventional approaches with the advantages of low cost and portability. In this branch, TENG can be structurally divided into various working modes such as oscillating [10,11,12], swinging [13,14,15,16,17], and rotating [18,19,20,21,22], and the rotary type has outstanding output performance due to its regularity and continuity. Among these modes, the rotational structure was a common way for TENG to harvest natural wind energy by fixing two or more electrode materials on the stator and rotor [22,23,24,25,26]. Unavoidably, the repeated friction between two dielectric materials during flutter motion poses challenges in the robustness and durability of the wind-driven TENGs and eventually causes wear failure. Currently, strategies for improving the device’s durability include noncontact mode [27,28,29], rolling friction [30,31], soft contact with the fur [15,32], and liquid lubrication [27,33,34,35]. Among those concepts, the rolling friction mode starts directly from the basic physical concept that the rolling wear is generally much higher than that of sliding; thus, high durability and sound outperformance may be simultaneously reached.

Polyvinylidene fluoride (PVDF) is widely used in the electrical manufacturing industry due to its excellent mechanical, thermal, and chemical properties [36,37,38,39,40]. PVDF film by electrospinning has been proven to be an excellent friction layer with a greater dielectric constant and a bigger negative charge [41]. However, its durability is hardly mentioned in other studies. As a result of the vulnerable interface, a more flexible friction strategy needs to be studied to adapt to the friction layer with excellent electrical performance. In this paper, we developed a durable and low-wear triboelectric nanogenerator for efficient energy harvesting. By utilizing PVDF composite nanofibers film as the triboelectric material, large specific surface area and softness in contact were simultaneously reached for improved output and durability. The ball-mill-inspired TENG (BMI-TENG) consists of a hollow cylinder mounted on a metallic frame and rotates around its longitudinal axis. The iron balls with composite nanofibers form triboelectrification electrode pair with the interdigital electrodes in the inner surface of the drum. When the drum rolls, the balls move from one copper electrode to another, and frictional electricity comes into being to form a non-contact collision mode between the balls due to electrostatic repulsion, and the reduced tangential force helps to protect the mechanical vulnerability of nanofibers. In addition, the durability is also accompanied by easy maintenance. The filler can be conveniently replaced and recycled after they reach the service life. The as-designed system attained enhanced robustness and a sound output performance, with an open-circuit voltage (Voc) of 272 V and a short circuit current (Isc) of 16 μA under the free-standing triboelectric-layer mode. The approximate linearity of Isc with rotation speed and wind speed shows the potential application in both distributed energy devices and self-powered rotation/wind speed monitoring sensors.

## 2. Experimental

### 2.1. Materials

PVDF (Polyvinylidene fluoride, HSV-900, Arkema Kynar, Paris, France), TPU (Polyester-based thermoplastic polyurethanes, BTE85AU, Evermore Chemical Industry Co., Ltd., Taiwan, China), DMF (N, N-Dimethylformamide, Aladdin D112000) and THF (Tetrahydrofuran, Aladdin T103266). All chemicals were used as received without further purification. Conductive rings were purchased from Moflon Technology Co., Ltd. (Shenzhen, China). An electronic pressure regulator was obtained from Jinglian Automation Co., Ltd. (Yueqing, China). Anemometer (AT186) was purchased from Smart Sensor (Hong Kong, China). 

### 2.2. Fabrication of the Composite Triboelectric-Layer

**Figure 1:**
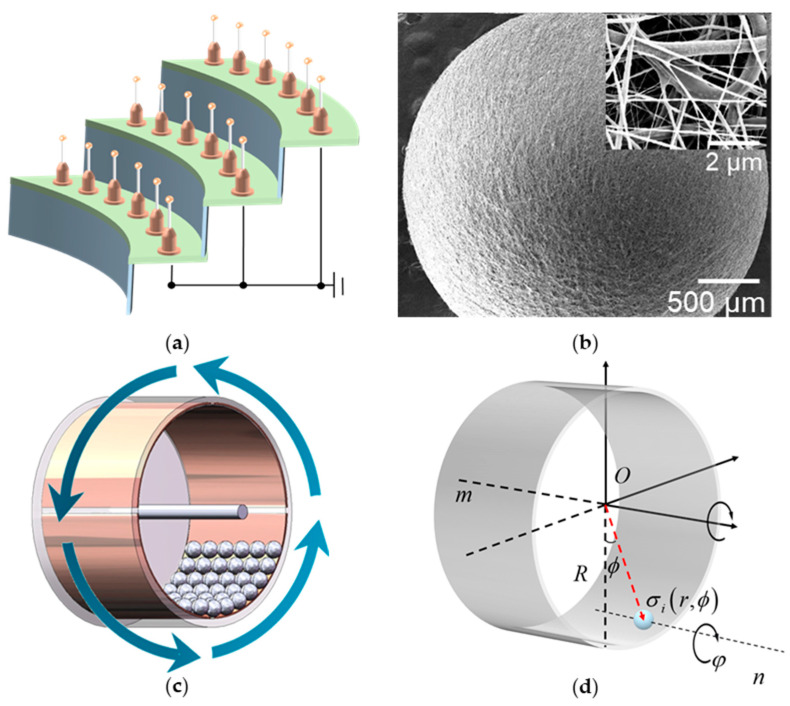
(**a**) Schematic diagram of nanofiber collector. (**b**) SEM images of nanofibers on the surface of the ball. (**c**) Structure of the BMI-TENG. (**d**) Analysis of single ball movement.

The production process is as follows. First, 10% ωt. mixture (PVDF:TPU = 4:1, by weight) was fully dissolved in organic dissolvent (DMF:THF = 1:1, by weight) and stirred for 6 h at room temperature, then poured into the syringe. A fibers collector was prepared by placing copper balls of different sizes at the heads of needles, which were neatly arranged on the acrylic plate neatly (Figure 1a). A high voltage of 10 kV was applied between the syringe and the collector, with 12 cm between them. Nanofibers were uniformly deposited on the surface of the metal balls under electrostatic force. In addition, a grounded metal plate was placed behind the collector to collect redundant fibers. The solution was electrospun for an hour at a rate of 0.8 mL/h to deposit a 50-micron film on the balls’ surface while the humidity and temperature were controlled at 45 ± 5% and 20 ± 2 °C. The surface of the balls was covered with a layer of well-distributed fibrous PVDF-TPU. Then, the balls were separated from the collector and put into a vacuum-drying oven for eight hours at 60 °C for the solvents to evaporate. Figure 1b shows the micromorphology of composite nanofibers under a Zeiss Merlin scanning electron microscope (Zeiss, Oberkochen, Germany). The thickness of the fiber film was about 0.1 mm.

### 2.3. Fabrication and Design of the Designed BMI-TENG

The design is inspired by a ball mill, and the BMI-TENG is fabricated with a free-standing triboelectric-layer mode, which is one of the four fundamental working modes of TENG. The even number of copper foils was divided into two opposite groups, pasted on the inner side of the drum as the collector, and the free-moving balls acted as the dielectric layer. Unlike the traditional free-standing triboelectric-layer mode, the dielectric layer of the device remains stationary, and the electrode contacts the dielectric layer alternately with the rotation of the drum. The dielectric layers and electrodes produce frictional charges when in contact, and electrons remain on the dielectric layer after separation. These electrons follow the dielectric layer and contact different electrodes alternately, which causes the electrons to flow between the two electrodes to balance the local potential distribution.

As shown in Figure 1b, the nanofiber layer prepared by electrospinning make endows the small ball with a large specific surface area. The low-wear contact mode proposed in this design improves the application range of nanofibers in nanogenerators. In addition, polyester-based TPU was doped in pure PVDF fibers, and TPU formed a solid network structure to protect the PVDF and reduce wear loss. With the metal core and polymer shell, the spherical dielectric layer generates and carries frictional charges of higher density. The precise structure is shown in Figure 1c. The multifunctional energy harvesting device included a rotary drum and spherical microunit triboelectric layers. The drum consists of an acrylic tube (internal diameter = 74 mm, external diameter = 80 mm, length = 50 mm) and two round acrylic plates (diameter = 80 mm, thickness = 2 mm). Even pieces of the Cu electrodes were pasted on the inner side of the acrylic tube, which was divided into two groups of symmetric electrodes. Suppose the balls move between two adjacent electrodes, which causes the electrons to flow between the two electrodes to balance the local potential distribution. As a shaft, a thinner acrylic rod (diameter = 5 mm, length = 100 mm) passed through the center of the acrylic plates and conductive ring, which connected with Cu electrodes. The electrons accumulated on the electrode were exported to the external circuit through the brush inside the conductive ring.

Unlike traditional rotational TENG, there is no stator in the structure of BMI-TENG. Relative motion between friction layers is generated under the constraint of the gravity field. The contact mode of the two friction layers is softer and more flexible, which reduces mechanical loss and prolongs the device’s service life. In addition, after the balls were charged, due to carrying the same kind of inductive charge, the balls repelled each other and had a non-contact collision, which helped solve the mutual friction between balls. In addition, the durability is accompanied by easy maintenance, and the filters can be conveniently replaced and recycled after they reach their service life.

## 3. Results and Discussion

### 3.1. Working Principle

**Figure 2:**
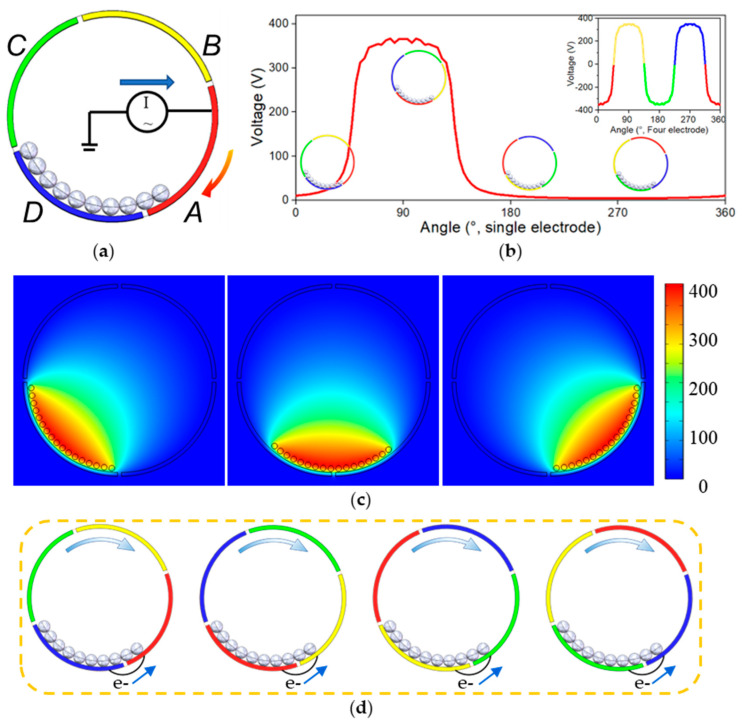
(**a**,**b**) Potential diagrams of TENGs with single and four electrodes, respectively. (**c**) Potential distribution for different ball positions using COMSOL. (**d**) Schematic diagram of electronic flow.

The working principle of TENG can be divided into two parts. As shown in Figure 2a,b, the rolling contact between the ball and the copper electrode causes the ball to be charged. The charge distribution on the surface of each sphere is uneven, and the polarized metal core balances the charge distribution. In the second part, the continuous movement of the balls between different electrodes causes the electrodes to form a constantly changing electric field to balance the static electricity inside the drum. When the drum rotated, the gravity field kept the balls at the bottom of the drum. In addition, due to the rotation of the drum, there would be a deviation in the direction of rotation. The balls could be regarded as moving along the inner side of the drum so that the motion state of the ball could be decomposed into the rotation of the barycenter along the drum’s axis and rotation on its own axis (Figure 1d). The motion process could be described by the following equations about the axis of the drum and ball separately. If the drum is chosen as the reference system, the balls could be regarded as the circular motion described by Equation (1). This means the balls could move from one electrode to the next because of the drum’s rotation. AC output is produced because of the revolutions. All terms entering following equations are explains in Table 1.

(1) $$I_{im}\frac{d^{2}\phi }{dt^{2}}=M_{im}\left(t\right)=G_{i}R\sin\phi +F_{e,im}\left(t\right)R+F_{c,i}\left(t\right)R$$

(2) $$I_{in}\frac{d^{2}\varphi }{dt^{2}}=M_{in}\left(t\right)=F_{e,im}\left(t\right)r+F_{c,i}\left(t\right)r$$

Considering TENG with only one electrode, the output signal is shown in Figure 2b. When the drum rotates, the charged ball gradually moves to the red electrode, increasing the induced charge. When all the balls stay on the red electrode, the induced charge is the most, and the voltage signal reaches the maximum. As the rotation keeps working, the balls leave the red electrode, and the signal gradually decreases until it disappears.

A theoretical study of TENG with a single electrode has been carried out using the finite element method in COMSOL software (Figure 2c). The induced electric field of the electrode is related to the motion equation of the ball. When the ball enters above the electrode, the electrode starts to generate an induced electric field. The induced electric field disappears when the ball is far away from the electrode.

(3) $$V_{A}\Rightarrow f\left(\phi \right)$$

The electric potential distribution under open-circuit conditions was calculated for different ball positions over the Cu electrodes. The motion of a single 3 mm diameter ball rolling on the two consecutive electrodes was evaluated. To clarify the device’s working principle, the charge distribution was calculated when the ball moved from one electrode to the next.

If there are four electrodes (A, B, C, D) alternately connected to two output ports (Figure 1a), the device would generate forward and reverse pulse currents in turn and could continuously produce output AC signals in each time cycle,

(4) $$\begin{array}{cc} V_{oc}\left(\phi \right) & =V_{A}\left(\phi \right)-V_{B}\left(\phi \right)+V_{C}\left(\phi \right)-V_{D}\left(\phi \right)\\ & =V_{A}\left(\phi \right)-V_{A}\left(\phi +\frac{\pi }{2}\right)+V_{A}\left(\phi +\pi \right)-V_{A}\left(\phi +\frac{3\pi }{2}\right) \end{array}$$

When *n* balls are in the device, the output signal is the superposition of *n* signals.

(5) $$U_{oc}\left(\phi \right)=V_{oc}\left(\phi \right)+V_{oc}\left(\phi +\delta \right)+V_{oc}\left(\phi +2\delta \right)\ldots +V_{oc}\left(\phi +n\delta \right)$$

Figure 2d shows the continuous transfer of electrons between electrodes.

Furthermore, using a low-speed motor as an excitation source and an electrometer (6514, Keithley, Cleveland, OH, USA) as the output signal receiver, the essential performance of the BMI-TENG was investigated to verify our view (Figure 2e,f). It is worth mentioning that the voltage increased gradually at the beginning, which meant that the friction charges accumulated on the surface of the balls were growing. After several cycles, the amplitude of the voltage was almost constant. Owing to the rotation of balls (Equation (2)), every part of the ball’s surface was in effective contact with the copper electrode. The device forms a 3 mm thick three-dimensional mesh friction layer with a small ball as the carrier, plus a nanofiber structure, which improves the effective contact area in the macro and micro aspects separately.

### 3.2. Measurement and Device Optimization

**Figure 3:**
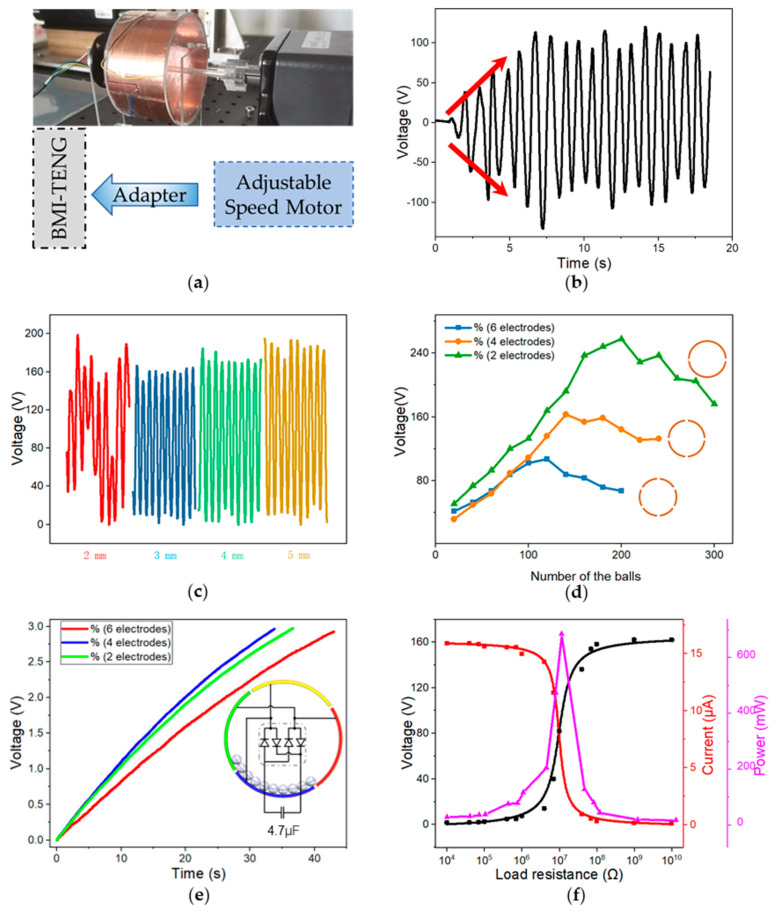
(**a**) Schematic diagram of mock test. (**b**) Charge-accumulating process of the device at 50 rpm. (**c**) The output of the TENG with different-diameter balls. (**d**) Effect of the number of electrodes and balls on output. (**e**) TENG charged the capacitors. (**f**) Load resistance dependency of the power of TENG.

The diameter and weight of the balls play a vital role in charge transfer efficiency. Balls with different diameters were divided into four groups for the experiment. To control the variables, the balls of each group occupied the same area. The results show that the experimental results corresponding to the most miniature ball were unsatisfactory (Figure 3a). As a result of electrostatic adsorption during rotation, the petite balls clung to the electrodes tightly and significantly reduced the charge transfer efficiency with the decrease in size. After 2000 cycles of operations of the four groups of devices, the balls were taken out and examined under the scanning electron microscope. The nanofibers on the surface of 4 mm and 5 mm balls curled up badly.

Meanwhile, the performance of the device with 4 mm and 5 mm balls was seriously reduced (>20%), and the device with 3 mm balls was only reduced by about 10%. This meant that the small ball had an intense collision with the electrode during the movement, and the balls with large diameters had a greater electrostatic repulsion during the collision, which reduced wear and made the nanofibers on the surface of the larger balls less vulnerable. Therefore, balls of 3 mm were more suitable for this device and used for the later test.

In order to study the influence of the number of balls on different devices, the number of balls was replaced with the filling rate, which indicates the portion of the dielectric layer area covered by the balls to the whole extent of the dielectric layer. Theoretically, more charge was carried with more balls. However, if there were too many balls to cover two adjacent electrodes, which carry opposite charges, the induced charges would offset each other. Therefore, it is appropriate that the balls could just cover a certain part of the electrode surface. Another consideration was that dynamic balls would collide with each other, which would occupy more space than static balls, especially when the drum spun at high speed. In other words, the balls did not actually need to cover the entire electrode when the drum was motionless, and the ball should cover the whole electrode when the drum rotated. The statistics in Figure 3b offered further support for our theory. When 2, 4, and 6 electrodes were used, the BMI-TENG had the highest output when the filling rate was 34.2%, 51.2%, and 56.3%, respectively. The arc angle of the electrode layer exceeded 180° with two electrodes, and the balls were stacked together. In this case, the filling rate has no reference significance. Therefore, about 50–60% of the filling rate is appropriate for the electrode layer area in the current case.

The number of electrodes is also the core parameter of BMI-TENG. The device with a double number of electrodes means the area of a single electrode also doubles. While doubling the output cycles, the output power of a single cycle significantly decreases. In order to find out the effect of the number of electrodes on the charge transfer efficiency, the rectifier bridge was connected to the output of the device with a different number of electrodes. It was found that the device with two electrodes produced the highest open circuit voltage (257 v), and the device with four electrodes charged the capacitor the fastest (Figure 3c), which is more suitable for energy collection. The energy-harvesting capability of BMI-TENG was investigated by calculating the output power. When the load resistance was 10 MΩ, the instantaneous output power of a single BMI-TENG unit reached 685 μW (Figure 3d).

**Figure 4:**
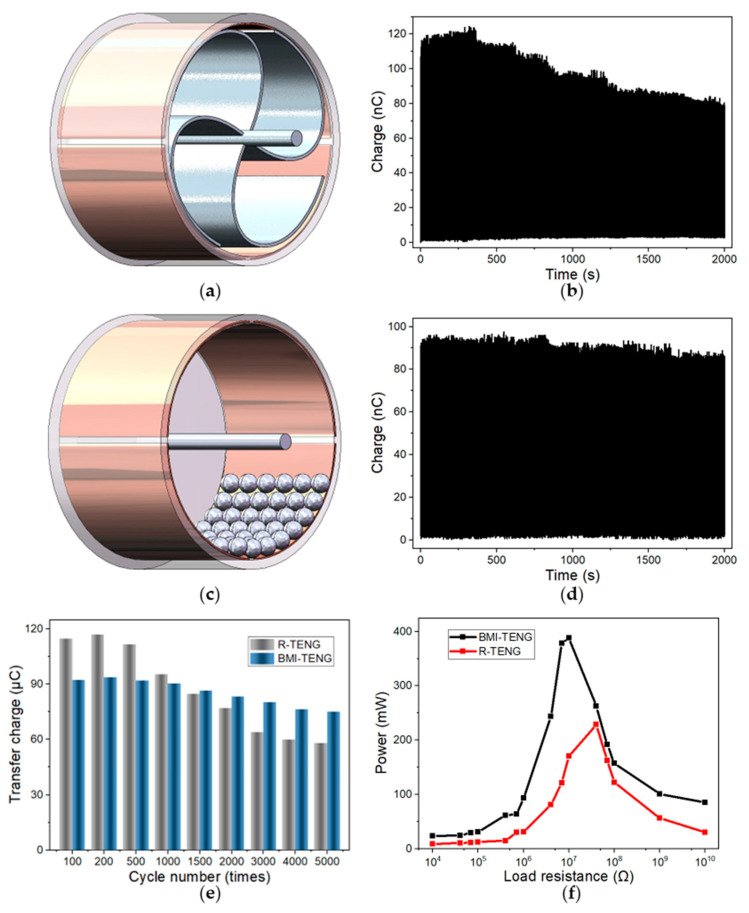
Structure of R-TENG (**a**) and BMI-TENG (**c**). Transferred charge curve of 2000 cycles of the R-TENG (**b**) and BMI-TENG (**d**). (**e**) Comparison of long-term performance (5000 cycles). (**f**) Output power of TENG with various resistances after 5000 cycles.

Nanofiber film has an excellent performance in the field of triboelectricity, and the unique structure endows the friction layer with high charge density. However, its poor mechanical properties limit its application in triboelectric nanogenerators. To verify the superiority of BMI-TENG and the traditional rotation TENG (R-TENG) in mechanical loss, two devices were made for comparison with friction layers of equal area and equal thickness, and the structure was shown in Figure 4a,c. Although it is more flexible to use the balls as the friction layer, not all nanofibers can contact the copper electrode at the same time due to the three-dimensional structure of the balls. In the first 1200 cycles of our control experiment, R-TENG performed better, transferring more charge in each cycle. However, the output performance of R-TENG declined faster than that of BMI-TENG. After 1200 cycles, the performance of the two groups of devices became equal, and R-TENG had lost its advantages in energy conversion. After 2000 cycles, the charge transfer of the R-TENG decreased significantly (34.1%) due to the severe wear of the film. BMI-TENG had only 11.0% attenuation. In order to verify the reliability of the equipment in long-term operation, we recorded and compared the transfer charge in 5000 cycles (Figure 4e). The load power test was carried out with two TENG after 5000 cycles of operation (Figure 4f). The results show that BMI-TENG performance is almost stable and better than R-TENG. Our proposed structural optimization reduced material wear by more than twice after 5000 cycles (BMI-TENG: 19.87%; R-TENG: 46.45%).

Obviously, the rotary-drum structure is successful in improving the durability of TENG due to the low wear that benefits from the rolling mode and electrostatic repulsion among the rolling fillers. Finally, R-TENG transferred more charge than BMI-TENG in the beginning, but BMI-TENG performed better in the long run, which was more suitable for outdoor distributed self-driving sensors.

### 3.3. Performance and Applications

**Figure 5:**
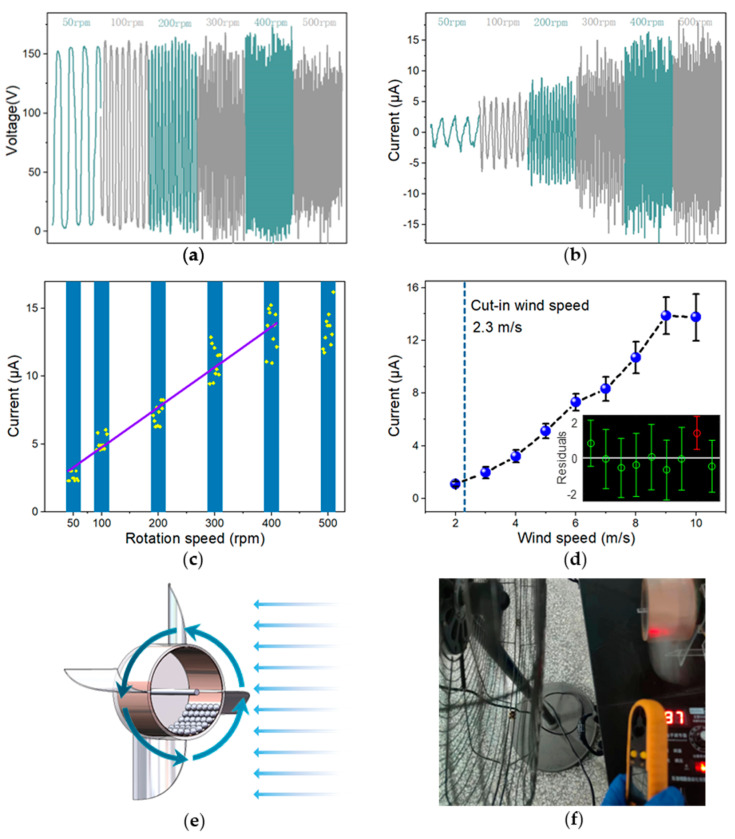
(**a**,**b**) Voc and Isc of the BMI-TENG change along with the rotation speeds. (**c**,**d**) Linear fitting results and residual analysis of the output current signals (Isc) of the BMI-TENG with rotating speed and the wind speed range from 2 to 10 m/s. (**e**) Structure diagram of wind energy collector. (**f**) Wind speed dependency test.

In order to simulate the performance of BMI-TENG under various rotation speeds, excitation at different rates was applied to the equipment through low-speed motors. As the engine speeded up, the open circuit voltage remained constant, whereas the short circuit current increased (Figure 5a,b). In addition, an approximately linear relationship between short-circuit current and speed within 400 rpm was also found (Figure 5c).

(6) $$I_{\mathrm{sc}}=0.0296\cdot RS+1.7894\;\left(RS\in \left[50,400\right]\right)$$

Here, *I*_sc_ is the short circuit current, and *RS* is the rotation speed (RPM). That means BMI-TENG has potential application prospects for speed measurement below 400 rpm. As the rate increased continuously, the output fluctuation became volatile. This phenomenon resulted from the fact that with the increase of the centrifugal force, some of the balls rotated with the drum and no longer transferred charge. Therefore, the BMI-TENG is more suitable for energy collection at low rotation speeds, such as collecting energy from water flow and natural wind while providing accurate rolling for monitoring wind speed.

A wind energy collector was assembled by BMI-TENG and four fan blades. Figure 5e,f shows four curved blades installed on the side of the TENG and drove the rotation of BMI-TENG in the wind farm. Different wind farms were provided with high-power fans with electronic pressure regulators, and wind speeds were tested with an anemometer. Figure 5d shows the change in the output current of the generator along with the wind speed. It is worth mentioning that the cut-in wind speed is about 2.3 m/s. However, after the wind speed dropped to 2 m/s, the device could still work continuously. As the wind speed increased, the peak average current of the device also increased. The maximum output current can achieve 13.5 µA at the wind speed of 10 m/s. The relationship between the wind speed and the current can be obtained by linear fitting, and the wind speed sensitivity is about 1.74 μA·s·m^−1^.

(7) $$I_{\mathrm{sc}}=1.7400\cdot WS-3.1853\;\left(WS\in \left[2,10\right]\right)$$

Here, *WS* is the wind speed (m/s). R^2^ is the correlation coefficient, and the accuracy of fitting results is 97.97%, and the regression model is more consistent with the original data when the wind speed range is 2 to 8 m/s. The regression model is rebuilt within this range:

(8) $$I_{\mathrm{sc}}=1.6260\cdot WS-2.7459\;\left(WS\in \left[2,8\right]\right)$$

The sensitivity of wind speed is about 1.63 μA·s·m^−1^, and the accuracy of fitting results is 98.49%, demonstrating the potential of the as-designed BMI-TENG for wind speed monitoring.

**Figure 6:**
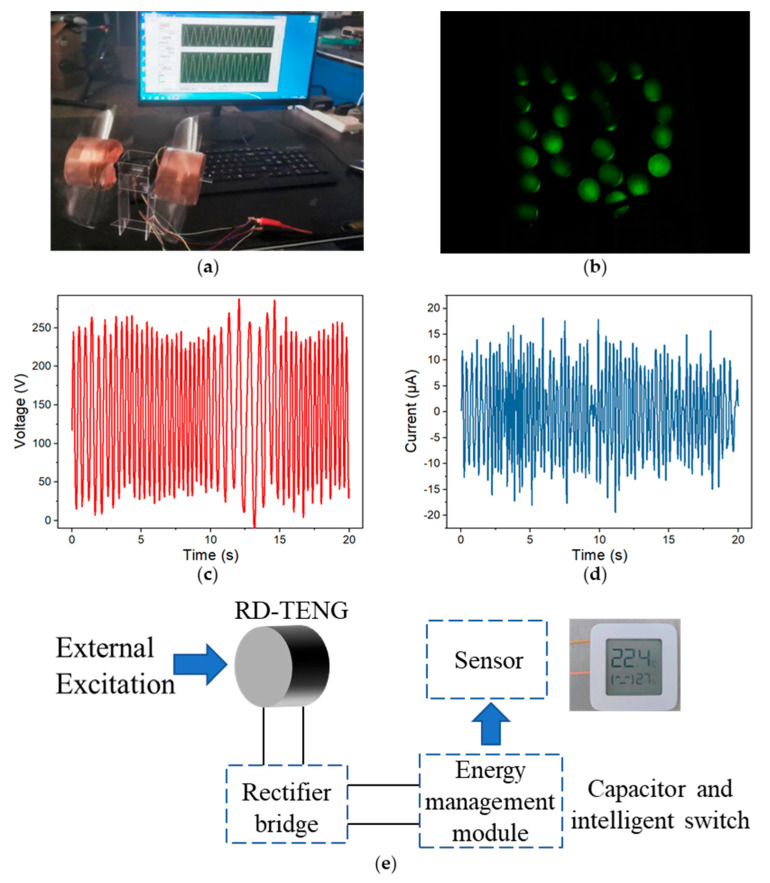
(**a**) BMI-TENG installed on the windmill. (**b**) Photograph of LEDs lit directly by the BMI-TENG with an “RD” pattern. (**c**,**d**) The output of collecting natural wind energy. (**e**) Schematic diagram of the integrated self-powered sensor.

Two BMI-TENGs on the windmill with blades made of acrylic by laser cutting were installed (Figure 6a). Figure 6b shows the device can directly light 24 LEDs. The open circuit voltage (1.7 times) and short circuit current (1.9 times) of the system composed of two BMI-TENGs were significantly increased, which showed that combining multiple power generation units together is feasible to gain higher power output (Figure 6c,d). Then, the energy management module was installed on the base of the windmill, including capacitors and intelligent switches. The generator charges the capacitor to 3 volts to power small electronic devices. Figure 6e shows the successful driving of the temperature and humidity sensor. Moreover, its fully sealed structure can protect the power generation unit from the negative impact of the external environment, such as moisture, making the device practicable for outdoor energy collection.

## 4. Conclusions

In summary, a ball-mill-inspired durable TENG was proposed and designed for wind energy harvesting. By electrospinning a layer of composite nanofibers on the filler balls, mechanical loss can be decreased by the rolling mode and electrostatic repulsion among the fillers. After prolonged periods of operation, the device is able to maintain 80% of its electrical performance. Moreover, it represents a significant improvement over traditional structures by reducing wear by more than two-fold. As a result, the BMI-TENG achieves a long service life, high durability, and easy maintenance. By assembling multiple BMI-TENGs, natural wind energy can be efficiently harvested to drive portable electronics such as temperature and humidity sensors. Based on the high sensitivity and wide linearity of the rotation, a self-powered wind speed sensor has been demonstrated, which presents a cost-effective, sustainable, and easily maintained power source and wind speed sensor for various WSN (Wireless Sensor Network) applications, such as climate monitoring and environmental fire detection.

## Figures and Tables

**Table 1:** Terminology of ball-motion model.

Term	Explanation
*R*	Radius of the drum
*r*	Radius of the ball
*I_im_*	Moment of inertia about the m-axis
*I_in_*	Moment of inertia about the n-axis
*M_im_*	Rotational torque about m-axis
*M_in_*	Rotational torque about n-axis
*ϕ*	Rotation angle of the drum
*φ*	Rotation angle of the ball
*G_i_*	Gravity of the ball
*F_e,im_*	Electrostatic force about m-axis
*F_e,in_*	Electrostatic force about n-axis
*F_c,i_*	Contact force

## Data Availability

No new data were created or where data is unavailable due to privacy or ethical restrictions.
